# Clinical outcomes of carbapenem therapy in OXA-48–producing Enterobacterales infections: a French multicentre cohort, systematic review, and meta-analysis

**DOI:** 10.1080/22221751.2026.2671518

**Published:** 2026-05-07

**Authors:** Laurent Dortet, Charlotte Moreau, Aurélien Dinh, Rémy A. Bonnin, Lélia Escaut, Manon Seguret, Laura Weber, Samy Figueiredo, Benoît Pilmis

**Affiliations:** a Team “Resist” UMR1184 “Immunology of Viral, Auto-Immune, Hematological, and Bacterial diseases (IMVA-HB).” INSERM, CEA, Faculty of Medicine, Université Paris-Saclay, Le Kremlin-Bicêtre, France; b Department of Bacteriology-Hygiene, Bicêtre Hospital, Assistance Publique–Hôpitaux de Paris, Faculty of Medicine and Paris-Sud University, Le Kremlin-Bicêtre, France; c Associated French National Reference Center for Antibiotic Resistance: Carbapenemase-Producing Enterobacterales, Le Kremlin-Bicêtre, France; d SEPSIS Comprehensive Center – IHU SEPSIS, France; e Department of Infectious Diseases and Tropical Diseases, Assistance Publique–Hôpitaux de Paris, Raymond Poincaré Hospital, Paris-Saclay University, Boulogne Billancourt, France; f Department of Infectious Diseases and Tropical Diseases, Assistance Publique–Hôpitaux de Paris, Bicêtre Hospital, Paris-Saclay University, Le Kremlin-Bicêtre, France; g Department of Anesthesia and Surgical Intensive Care, Bicêtre Hospital, Assistance Publique–Hôpitaux de Paris, Paris-Saclay University, Paris, France; h Department of Clinical Microbiology, Saint-Joseph & Marie-Lannelongue Hospitals, Paris, France

**Keywords:** OXA-48, carbapenemase-producing Enterobacterales, carbapenem therapy, ceftazidime-avibactam, antimicrobial resistance, meta-analysis

## Abstract

OXA-48–producing Enterobacterales (OXA-48-PE) represent a growing global health threat. Most of OXA-48–PE remain categorized susceptible to carbapenems, which might encourage their use despite uncertain clinical efficacy. This study evaluated clinical outcomes of infections caused by OXA-48-PE treated with carbapenem compared with alternative active therapies. The analyses were performed at three levels: a descriptive analysis of the French cohort, a comparative descriptive analysis of all published studies, and a meta-analysis restricted to studies providing direct comparisons between carbapenem-based and alternative active regimens. Between September 2021 and March 2023, 59 patients with monomicrobial OXA-48-PE infections were included in a French multicenter retrospective cohort. In parallel, a systematic review was conducted to identify clinical studies published through 31 December 2024, reporting outcomes of OXA-48-PE infections. In the French cohort, the overall 30-day mortality was 49.1%. Clinical failure occurred in 57.1% of patients receiving meropenem monotherapy, including patients infected with isolates exhibiting meropenem MICs within the susceptible range. Across 12 clinical studies (817 patients), carbapenem therapy was associated with high and variable crude mortality (52%), whereas newer agents active against OXA-48-PE, particularly ceftazidime-avibactam, were associated with lower and more consistent crude mortality (30.7%). In the primary meta-analysis of 6 human comparative studies, carbapenem therapy was associated with an increased risk of clinical failure compared with alternative active regimens (OR = 2.02; 95% CI = 1.05–3.88). Despite apparent *in vitro* susceptibility, carbapenem therapy was consistently associated with unfavourable clinical outcomes in OXA-48–PE infections, supporting the prioritization of alternative active agents whenever available.

## Introduction

Carbapenem-resistant Enterobacterales (CRE) are recognized as a major global health threat and are classified by the World Health Organization as critical priority pathogens [1]. Among CRE, carbapenemase-producing Enterobacterales (CPE) represent a particular challenge because of their limited therapeutic options and rapid international dissemination. In Europe and Mediterranean countries, OXA-48-like carbapenemases have become predominant, creating major therapeutic dilemmas [2]. Unlike other carbapenemases, OXA-48-like enzymes often confer only low-level resistance to carbapenems, resulting in minimum inhibitory concentrations (MICs) that frequently fall within the susceptible range according to European Committee on Antimicrobial Susceptibility Testing (EUCAST) breakpoints. Consequently, carbapenems – particularly meropenem – are still used for infections caused by OXA-48-PE, despite ongoing uncertainty regarding their clinical efficacy.

The microbiological categorization contrasts with recommendations from infectious diseases societies, including the European Society of Clinical Microbiology and Infectious Diseases (ESCMID) and the Infectious Diseases Society of America (IDSA), which discourage carbapenem monotherapy for carbapenemase-producing Enterobacterales [3,4]. Experimental data further question the reliability of MIC-based susceptibility interpretation: in a murine pneumonia model, meropenem failed against an OXA-48-producing *Klebsiella pneumoniae* strain with a low meropenem MIC, highlighting the potential disconnect between *in vitro* susceptibility and *in vivo* response [5].

The introduction of novel β-lactam/β-lactamase inhibitor combinations active against OXA-48-PE, such as ceftazidime-avibactam and emerging agents including cefepime-based inhibitor combinations [6,7] and cefiderocol, has expanded therapeutic options, although access remains uneven worldwide. However, the overall clinical impact of carbapenem therapy compared with alternative active regimens in OXA-48-PE infections remains insufficiently defined, with available evidence largely derived from heterogeneous observational studies.

To address this gap, we conducted a three-level investigation. First, we analyzed a French multicenter cohort to identify preliminary clinical evidence in routine practice. Second, we performed a systematic review with comparative descriptive analysis in order to capture the full breadth of the available clinical literature, including heterogeneous studies that were not suitable for pooled comparative effect estimation. Third, we conducted a restricted meta-analysis limited to studies providing direct comparisons between carbapenem-based regimens and alternative active therapies, in order to obtain a more interpretable quantitative estimate. This integrated framework was designed not to rely on a single retrospective cohort alone, but to clarify a clinically important question that remains insufficiently resolved in the literature: whether carbapenem therapy can be considered reliable in OXA-48-producing Enterobacterales infections despite apparently susceptible MIC values.

## Patients and methods

### Study design and overall framework

This work was designed as a three-level investigation to evaluate the clinical impact of carbapenem therapy compared with alternative active regimens in infections caused by OXA-48–PE.

The first level consisted of a French multicenter retrospective cohort study aimed at describing clinical outcomes and identifying preliminary clinical evidence. The second level consisted of a systematic review with comparative descriptive analysis of all available clinical studies, in order to summarize the broader literature, including heterogeneous studies that were not suitable for pooled comparative effect estimation. The third level consisted of a restricted meta-analysis limited to studies providing direct comparisons between carbapenem-based regimens and alternative active therapies, in order to generate a more interpretable quantitative estimate.

Each component was conducted and analyzed separately, with predefined objectives and methodological approaches, as detailed below.

### Part 1 – French multicenter retrospective cohort study

#### Study design and setting

We conducted a French multicenter, observational, retrospective cohort study between September 2021 and March 2023. Patients were identified through the Associated French National Reference Center for Antibiotic Resistance for carbapenemase-producing Enterobacterales.

#### Patient selection

Adult patients (≥18 years) were eligible if they had a documented monomicrobial infection caused by an OXA-48-PE. Patients were excluded if they had colonization without clinical infection, polymicrobial infections, missing data on antibiotic therapy, or unavailable outcome data.

#### Definitions and data collection

Infections were classified according to the Centers for Disease Control and Prevention/National Healthcare Safety Network (CDC/NHSN) criteria and included bloodstream infections, urinary tract infections, intra-abdominal infections, respiratory tract infections, catheter related infections, skin and soft tissue infections, and other clinically documented infection sites.

Clinical and demographic data were extracted from medical records, including age, sex, comorbidities (Charlson Comorbidity Index), hospitalization setting, intensive care unit admission, and presence of septic shock at infection onset. Microbiological data included bacterial species identification, antimicrobial susceptibility testing, and minimum inhibitory concentrations (MICs), interpreted according to EUCAST criteria in use at the time of the study.

#### Treatment classification and outcomes

Definitive antibiotic therapy was categorized into carbapenem monotherapy (imipenem or meropenem) or alternative active regimens. In the descriptive presentation of treatment groups, ceftazidime-avibactam was considered separately from other active non-carbapenem alternatives because of its distinct mechanism of action and more consistent activity against OXA-48–PE. Appropriateness of therapy was assessed by an infectious diseases specialist and a microbiologist based on MIC values and susceptibility profiles.

Clinical outcome was assessed 30 days after infection onset. The primary outcome was 30-day all-cause mortality. Because of the limited sample size and the small number of patients in the carbapenem group, comparisons between treatment groups were considered exploratory. Baseline comparisons were descriptive, and Fisher’s exact tests were performed only for selected categorical comparisons, with cautious interpretation because of limited statistical power. In addition, exploratory descriptive analyses of 30-day mortality were performed according to infection source and treatment group.

#### Ethical considerations

The study was conducted in accordance with the Declaration of Helsinki and was approved by the local ethics committee (approval no. 2022-0303). All patients received written information, and data were anonymized prior to analysis.

### Part 2 – systematic review and comparative descriptive analysis

#### Search strategy and study selection

A systematic literature search was performed in PubMed, Embase, and Web of Science to identify studies published up to 31 December 2024. The search combined keywords and controlled vocabulary related to OXA-48-PE, carbapenem therapy, alternative antimicrobial agents, and clinical outcomes. The full search strategies are provided in Supplementary Table S1.

Eligible studies included randomized controlled trials, prospective or retrospective cohort studies, and case series reporting clinical outcomes of OXA-48-PE infections treated with carbapenem-based or alternative active regimens. Only studies published in English, French, or Spanish were considered. In vitro studies were excluded.

#### Data extraction and synthesis

Two investigators independently screened titles and abstracts, reviewed full texts for eligibility, and extracted data using a standardized form. Extracted variables included study design, country, patient characteristics, infection type, antimicrobial regimens, and reported outcomes.

For this analysis, treatment regimens were grouped into four predefined therapeutic classes: carbapenems; modern agents active against OXA-48-PE (including ceftazidime-avibactam, cefepime-enmetazobactam, cefepime-taniborbactam, and cefiderocol); colistin-based regimens; and other agents. When combination therapies were reported, regimens were categorized according to the main active agent or dominant therapeutic backbone described in the original study. Combinations containing ceftazidime-avibactam or another modern OXA-48-active agent were assigned to the corresponding modern-agent class, whereas colistin-containing combinations were classified as colistin-based when colistin represented the principal non-carbapenem backbone. This categorization was intended for descriptive synthesis and may remain imperfect when treatment descriptions were limited or heterogeneous across studies. Outcomes were summarized descriptively for each treatment class using crude study-level mortality data. This descriptive synthesis was intended to provide a cross-study overview of outcome patterns rather than a pooled comparative effect estimate. Given the heterogeneity of study designs, patient populations, infection sources, treatment strategies, and outcome definitions, no formal pooled effect estimates were calculated at this stage.

### Part 3 – meta-analysis of comparative studies

#### Eligibility criteria for meta-analysis

For the meta-analysis, we identified studies providing direct comparisons between a carbapenem-based regimen and an alternative active therapy in OXA-48-PE infections. The primary quantitative synthesis was restricted to human clinical studies. Because one experimental animal study provided directly comparable outcome data, it was not included in the primary clinical pooled analysis but was examined separately in sensitivity analysis as supportive experimental evidence.

#### Outcomes and statistical analysis

The primary outcome for the meta-analysis was clinical failure, defined for each comparative study according to the original study report. In the French cohort, clinical failure was defined as death from any cause by day 30. For the meta-analysis, the extracted endpoint corresponded to the closest study-level measure of overall treatment failure, generally including all-cause mortality and/or lack of clinical response according to each original definition. Mortality was used separately for descriptive comparisons across heterogeneous studies when failure was not consistently reported. Odds ratios (ORs) with 95% confidence intervals (CIs) were calculated for each study.

Pooled estimates were generated using both fixed-effects (Mantel–Haenszel) and random-effects (DerSimonian–Laird) models. Given the expected clinical and methodological heterogeneity across studies, the random-effects model was considered the primary estimate. Statistical heterogeneity was assessed using the I² statistic and Cochran’s Q test. No non-human experimental studies were included in the primary meta-analysis. Experimental animal data were examined separately and are discussed as supportive evidence rather than pooled with clinical studies. Results were graphically displayed using a conventional forest plot as the primary meta-analytic visualization.

#### Risk of bias assessment

Risk of bias in observational studies was assessed using the Newcastle–Ottawa Scale. For randomized controlled trials, the Cochrane Risk of Bias 2 tool was applied. Risk of bias assessments were performed independently by two investigators, with discrepancies resolved by consensus.

## Results

### Part 1 – French multicenter retrospective cohort study

#### Patient characteristics

Between September 2021 and March 2023, 59 adult patients with a documented monomicrobial infection caused by OXA-48–PE were included in the French multicenter retrospective cohort. Baseline patient characteristics are summarized in Table 1. The median age was 71 years (interquartile range [IQR], 59–76), and 42 patients (71.2%) were male. The median Charlson comorbidity index was 4 (range, 0–12), with cardiovascular disease (38.9%), cancer (30.5%), and diabetes mellitus (22.0%) being the most frequent underlying conditions.

**Table 1.** Patients’ characteristics.

Characteristics	Total (n = 59)
Male sex, n (%)	42 (71.2)
Age, median (min-max)	71 (59–76)
**Comorbidities, n (%)**	
Cardiovascular	23 (38.9)
Cancer	18 (30.5)
Diabetes mellitus	13 (22)
Immunodeficiency	6 (10.1)
Charlson score, median (min-max)	4 (0–12)
**Hospitalization department, n (%)**	
Medical ward	27 (45.7)
Surgical ward	12 (20.3)
Intensive care unit	20 (33.9)
***Source of infection,* n (%)**	
Urinary tract infection	13 (22.0)
Intra-abdominal infection	12 (20.3)
Respiratory tract infection	11 (18.6)
Catheter-related infection	8 (13.6)
Skin and soft tissue infection	7 (11.9)
Other	8 (13.6)
**Bacterial species identified, n (%)**	
*Klebsiella pneumoniae*	18 (30.5)
*Escherichia coli*	16 (27.1)
*Enterobacter cloacae complex*	13 (22.0)
*Citrobacter freundii*	7 (11.9)
Other Enterobacterales	5 (8.5)
**Definitive antibiotic therapy, n (%)**	
Ceftazidime-avibactam	28 (47.4)
Cefepime	11 (18.6)
Meropenem	8 (13.6)
Ceftazidime	6 (10.2)
Colistin	2 (3.4)
Other	4 (6.7)
Septic shock, n (%)	14 (23.7)
**Outcomes, n (%)**	
30-day mortality, n (%)	29 (49.1)
Mortality by treatment	
Ceftazidime-avibactam	12 (20.3)
Cefepime	6 (10.2)
Meropenem	5 (8.5)
Ceftazidime	3 (5.1)
Colistin	0 (0)
Others	3 (5.1)

One third of patients (33.9%) required admission to an intensive care unit, and septic shock at infection onset was reported in 14 cases (23.7%). The most common sources of infection were urinary tract (22.0%), intra-abdominal (20.3%), respiratory tract (18.6%), and catheter-related infections (13.6%).

The predominant causative organisms were *Klebsiella pneumoniae* (30.5%), *Escherichia coli* (27.1%), and the *Enterobacter cloacae* complex (22.0%). Baseline characteristics according to definitive treatment group are presented in Table 2. Patients treated with carbapenem monotherapy showed broadly comparable descriptive baseline characteristics to those receiving alternative active therapies, although the carbapenem group had numerically higher proportions of septic shock at infection onset (37.5% vs 21.6%) and ICU admission (37.5% vs 33.3%). Exploratory Fisher’s exact tests did not identify statistically significant differences for these severity-related variables (septic shock, *p* = 0.379; ICU admission, *p* = 1.000), although these comparisons were markedly underpowered.

**Table 2.** Baseline characteristics according to definitive treatment group in the French cohort.

Variable	Total (n = 59)	Carbapenem monotherapy (n = 8)	Ceftazidime-avibactam (n = 28)	Other alternatives (n = 23)	All alternative active therapy pooled (n = 51)
Age, median [IQR]	71 [59–76]	61 [54–81]	78 [64–83.5]	73 [61–80]	74 [62–81]
Male sex, n (%)	42 (71.2)	4 (50)	20 (71.4)	18 (78.3)	38 (74.5)
Charlson score, median [IQR]	4 [2–5]	3 [2–5]	4 [3–5]	4 [2–5]	4 [2–5]
ICU admission, n (%)	20 (33.9)	3 (37.5)	11 (39.3)	6 (26.1)	17 (33.3)
Septic shock at infection onset, n (%)	14 (23.7)	3 (37.5)	7 (25)	4 (17.4)	11 (21.6)
Source of infection, n (%)					
Urinary tract infection	13 (22.0)	2 (25.0)	8 (28.5)	3 (13.1)	11 (21.6)
Intra-abdominal infection	12 (20.3)	1 (12.5)	5 (17.8)	6 (26.1)	11 (21.6)
Respiratory tract infection	11 (18.6)	1 (12.5)	4 (14.3)	6 (26.1)	10 (19.6)
Catheter-related infection	8 (13.6)	1 (12.5)	4 (14.3)	3 (13.1)	7 (13.7)
Skin and soft tissue infection	7 (11.9)	2 (25.0)	3 (10.7)	2 (8.7)	5 (9.8)
Other infection	8 (13.6)	1 (12.5)	4 (14.3)	3 (13.1)	7 (13.7)
Bacterial species identified, n (%)					
*Klebsiella pneumoniae*	18 (30.5)	4 (50.0)	8 (28.5)	6 (26.2)	14 (27.5)
*Escherichia coli*	16 (27.1)	2 (25.0)	7 (25)	7 (30.4)	14 (27.5)
*Enterobacter cloacae complex*	13 (22.0)	1 (12.5)	6 (21.4)	6 (26.2)	12 (23.5)
*Citrobacter freundii*	7 (11.9)	1 (12.5)	3 (10.7)	3 (13.1)	6 (11.7)
Other Enterobacterales	5 (8.5)	0 (0)	4 (14.3)	1 (4.3)	5 (9.8)
Appropriate definitive therapy, n (%)	54 (91.5)	7 (87.5)	28 (100)	19 (82.6)	47 (92.1)
30-day mortality, n (%)	29 (49.1)	5 (62.5)	12 (42.8)	12 (52.2)	24 (47.1)

#### Microbiological characteristics and meropenem MIC distribution

Antimicrobial susceptibility testing showed that the majority of OXA-48–PE displayed low meropenem minimum inhibitory concentrations (MICs). Overall, 83% of isolates had meropenem MICs ≤2 mg/L, corresponding to the susceptible-at-standard-dosing category according to EUCAST criteria. Within the overall cohort, MIC values were distributed as follows: ≤0.25 mg/L in 9 isolates (15.2%), 0.5 mg/L in 8 (13.6%), 1 mg/L in 14 (23.7%), and 2 mg/L in 18 (30.5%). Higher MIC values were less frequent, with 4 mg/L in 3 isolates (5.1%), 8 mg/L in 1 (1.7%), 16 mg/L in 3 (5.1%), and >16 mg/L in 3 (5.1%). Among patients treated with carbapenem monotherapy, meropenem MIC values were ≤0.25 mg/L in 3/8 isolates (37.5%), 0.5 mg/L in 1/8 (12.5%), and 2 mg/L in 3/8 (37.5%), indicating that carbapenem-treated patients were not limited to isolates at the upper boundary of the susceptible range. The distribution of meropenem MICs according to treatment regimen is shown in Supplementary Figure S1 and Supplementary Table S5. Notably, patients treated with carbapenem monotherapy were not limited to isolates with MICs at the upper boundary of the susceptible range, as low meropenem MIC values were also observed in this group.

#### Definitive antibiotic therapy

Definitive antibiotic regimens and clinical outcomes are detailed in Table 3. Among the 59 patients, 54 (91.5%) received an antibiotic regimen considered appropriate according to EUCAST susceptibility criteria, while 5 (8.5%) received inappropriate therapy.

**Table 3.** Treatment regimens and clinical outcomes in 59 patients with OXA-48-PE infections.

Treatment regimens	Subgroup	n/N	Failure rate (%)
Appropriate therapy (n = 54)	Total	25/54	46.2
	Meropenem (MIC ≤2 mg/L)	4/7	57.1
	Ceftazidime-avibactam	12/28	42.8
	Cefepime*	4/9	44.4
	Ceftazidime*	3/6	50
	Colistin	0/2	0
	Fluoroquinolones	1/1	100
	Trimethoprim-sulfamethoxazole	0/1	0
Inappropriate therapy (n = 5)	Total	5/5	100
	Meropenem (MIC > 8 mg/L)	1/1	100
	Cefepime	2/2	100
	Piperacillin-tazobactam	2/2	100

Data are number of failures per total patients (%). MIC, minimum inhibitory concentration.

Failure includes death and/or need for treatment modification

*The OXA-48-PE responsible for the infection did not produce any 3rd generation hydrolyzing enzyme (i.e. ESBL or AmpC).

Within the appropriate-therapy group, 7 patients received meropenem monotherapy for isolates with MICs ≤2 mg/L, and 47 received alternative active agents. Ceftazidime-avibactam was considered separately from the other alternative regimens and was by far the most frequently used alternative therapy (n = 28). The remaining alternatives were more heterogeneous and included cefepime (n = 9), ceftazidime (n = 6) in the absence of a co-produced third-generation cephalosporin-hydrolyzing enzyme, colistin (n = 2), and other agents including fluoroquinolones and trimethoprim–sulfamethoxazole.

#### Clinical outcomes

Overall, the 30-day mortality rate in the cohort was 49.1% (29/59). Clinical failure occurred in 25 of the 54 patients (46.2%) who received appropriate definitive therapy.

Among patients treated with meropenem monotherapy (MIC ≤ 2 mg/L), clinical failure was observed in 4 of 7 cases (57.1%). Descriptively, failure rates in patients treated with alternative active agents were 42.8% (12/28) with ceftazidime-avibactam, 44.4% (4/9) with cefepime, and 50.0% (3/6) with ceftazidime. No failures were observed among the two patients treated with colistin, while single cases treated with fluoroquinolones or trimethoprim–sulfamethoxazole showed variable outcomes (Table 3). Regarding 30-day mortality, carbapenem monotherapy was associated with a crude mortality of 62.5% (5/8), compared with 47.1% (24/51) for pooled alternative active therapies and 42.8% (12/28) for ceftazidime-avibactam. Exploratory Fisher’s exact tests were not statistically significant for carbapenem monotherapy versus pooled alternative active therapy (*p* = 0.472) or versus ceftazidime-avibactam (*p* = 0.434), and these comparisons should be interpreted with caution given the very limited sample size (Table 2). Exploratory descriptive analyses according to infection source also showed substantial heterogeneity in crude 30-day mortality across treatment groups. Among urinary tract infections, mortality was 50.0% (1/2) with carbapenem monotherapy versus 27.3% (3/11) with pooled alternative active therapies. Among intra-abdominal infections, the corresponding figures were 100.0% (1/1) versus 54.5% (6/11), whereas among respiratory tract infections mortality was 0/1 versus 60.0% (6/10). For catheter-related infections, mortality was 100.0% (1/1) versus 57.1% (4/7), for skin and soft tissue infections 50.0% (1/2) versus 60.0% (3/5), and for other infections 100.0% (1/1) versus 28.6% (2/7). These subgroup counts were small and are therefore presented descriptively only (Supplementary Table S3). Among the five patients who received inappropriate therapy, all experienced clinical failure (100%), including one patient treated with meropenem for an isolate with a meropenem MIC >8 mg/L. Importantly, clinical failures under meropenem therapy occurred despite low meropenem MICs in most cases, with 3 of 6 failures observed in patients infected with isolates categorized as susceptible according to EUCAST criteria.

#### Summary of findings from the French cohort

Taken together, this French multicenter retrospective cohort revealed a high overall rate of clinical failure and mortality among patients with OXA-48–PE infections. Although outcomes under meropenem therapy appeared unfavourable, these observations were based on a very limited number of carbapenem-treated patients. Exploratory formal comparisons did not demonstrate statistically significant differences, but these analyses were markedly underpowered and should not be interpreted as evidence of equivalence. Exploratory stratification by infection source also showed marked heterogeneity in crude mortality across subgroups, reinforcing the need for cautious interpretation of pooled cohort-level treatment comparisons. Accordingly, the cohort-level analyses were primarily intended to provide preliminary clinical context for the subsequent analyses of the published literature.

### Part 2 – systematic review and comparative descriptive analysis

This part of the analysis was intended to provide a descriptive overview of the available heterogeneous literature and was not designed to generate pooled comparative effect estimates; quantitative synthesis was reserved for the restricted meta-analysis of directly comparative studies.

#### Study selection and characteristics

The systematic literature search identified a total of 12 clinical studies reporting outcomes of infections caused by OXA-48–PE, in addition to the French multicenter cohort described above. These studies encompassed 817 patients and were conducted primarily in Europe and the Mediterranean region, including Spain, Turkey, France, Romania, and the United Arab Emirates.

Most studies were retrospective observational cohorts, with a smaller number of prospective cohorts and case series. Patient populations were generally composed of older adults, with median or mean ages ranging from 59 to 71 years, a predominance of male patients (55–70%), and high rates of critical illness, as reflected by intensive care unit admission rates ranging from 20% to 100% (Table 4).

**Table 4.** Summary of studies comparing clinical efficacy of carbapenems and alternative therapies against OXA-48-producing *Enterobacterales*.

Author (year)	Country	Study design	n	Median/mean age (years)	Male (%)	ICU (%)	Main infection sites	30-day mortality (%)
Internal cohort (2025) [8]	France	Multicenter retrospective	59	71	71	34	BSI 100%; UTI 22%; IAI 20%; RTI 19%	49
Navarro (2013) [9]	Spain	Prospective cohort	40	67	60	20	BSI 100%	50
Dumlu (2024) [10]	Turkey	Retrospective multicenter	151	62	55	100	BSI 41%; VAP 31%; CRBSI 18%	29
Lumbreras (2024) [11]	Spain	Retrospective	76	70	65	22	BSI 100%	31
Lazar (2024) [12]	Romania	Retrospective	57	66	61	30	BSI 42%; UTI 28%	38
Önal (2023) [13]	Turkey	Retrospective ICU	42	64	58	100	BSI 57%; VAP 43%	52
Aslan (2022) [14]	Turkey	Retrospective	124	62	53	57	BSI 100%	52
Corbella (2022) [15]	Spain	Retrospective	117	68	60	20	UTI 44%; RTI 10%; IAI 9%; CRBSI 10%	28
Rodríguez (2021)[16]	Spain	Retrospective	88	NA	70	36	UTI 39%; CRBSI 26%; RTI 16%	30
De la Calle (2019) [17]	Spain	Retrospective	23	59	83	33	IAI 29%; UTI 25%; RTI 21%	8
Ahn (2015) [18]	UAE	Case series	4	57	25	50	BSI 100%	25
Balkan (2014) [19]	Turkey	Retrospective	36	55	50	72	BSI 100%	50

CRBSI: Catheter-related bloodstream infection, IAI: Intra abdominal infection, ICU: intensive care unit, RTI: respiratory tract infection, UTI: urinary tract infection, VAP: ventilator associated pneumonia.

*Klebsiella pneumoniae* was the predominant pathogen across all studies, followed by *Escherichia coli* and members of the *Enterobacter cloacae* complex. Bloodstream infections were the most frequently reported infection type, followed by urinary tract, respiratory tract, and intra-abdominal infections.

#### Distribution of treatment strategies

Across the included studies, a wide variety of antimicrobial regimens were used. For the purpose of comparative descriptive analysis, treatments were grouped into four predefined therapeutic classes: (i) carbapenems; (ii) modern agents with activity against OXA-48–PE, including ceftazidime-avibactam, cefepime-enmetazobactam, cefepime-taniborbactam, and cefiderocol; (iii) colistin-based regimens; and (iv) other non-carbapenem active agents, including tigecycline, aminoglycosides, fluoroquinolones, fosfomycin, and trimethoprim–sulfamethoxazole. Carbapenem therapy was reported in most studies, either as monotherapy or as the primary comparator, reflecting its continued widespread use in clinical practice. Novel β-lactam/β-lactamase inhibitor combinations, particularly ceftazidime-avibactam, were increasingly represented in more recent studies, whereas colistin-based regimens and older agents predominated in earlier cohorts. Within the class of modern OXA-48-active agents, ceftazidime-avibactam accounted for most treated patients and was therefore emphasized separately in the descriptive interpretation whenever possible. Details of alternative regimens used in directly comparative studies are provided in Supplementary Table S4.

#### Meropenem MIC distribution in published studies

Among patients treated with carbapenem monotherapy, meropenem MIC data were available for 110 isolates. The vast majority of these isolates exhibited low meropenem MICs, with 91.8% having MIC values ≤0.5 mg/L. Intermediate MICs (0.5–2 mg/L), classified as susceptible at standard dosing according to EUCAST criteria, accounted for 6.3% of isolates, while high-level resistance (MIC >8 mg/L) was observed in only 1.9% of cases.

No isolates fell within the 2–8 mg/L MIC range corresponding to the “susceptible at increased exposure” category. The distribution of meropenem MICs according to treatment regimen is illustrated in Supplementary Figure S2, showing that carbapenem therapy was predominantly used in patients infected with isolates categorized as susceptible by EUCAST 2025 guidelines.

#### Mortality outcomes by therapeutic class

Mortality outcomes varied widely across studies and treatment classes. Among patients treated with carbapenems, reported mortality rates ranged from 25% to 100%, with an overall crude mortality of approximately 52% when excluding experimental animal studies.

In contrast, treatment with novel agents active against OXA-48-PE was associated with lower and more consistent mortality rates. Across ten studies including 483 patients treated with ceftazidime-avibactam, overall crude mortality was 30.7%, with reported values ranging from 8.3% to 54.8%. Newer agents such as cefepime–taniborbactam and cefiderocol were reported in small case series (≤10 patients) and were associated with survival in all reported cases.

Colistin-based regimens showed intermediate outcomes, with an overall crude mortality of 46.1% across 107 patients, but with marked variability between studies (range 0–100%). Other antimicrobial agents, including tigecycline, fosfomycin, aminoglycosides, fluoroquinolones, and trimethoprim–sulfamethoxazole, were associated with the poorest outcomes overall, with an overall crude mortality of 69.9% and a wide range of reported values.

**Figure 1.**
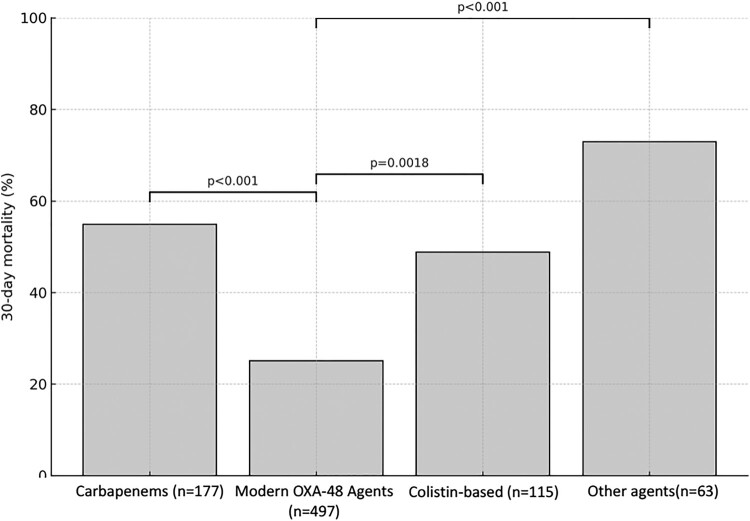
Descriptive cross-study summary of crude 30-day mortality according to treatment class in OXA-48–producing Enterobacterales infections.

Figure 1 provides a descriptive cross-study summary of crude 30-day mortality according to treatment class. In this descriptive overview, modern OXA-48-active agents, driven largely by ceftazidime-avibactam, were associated with lower crude mortality than carbapenem-based and colistin-based regimens. However, because the included studies differed substantially in patient populations, infection sources, treatment allocation, and outcome definitions, these between-class differences should be interpreted cautiously and not as formal comparative effect estimates.

Modern OXA-48-active agents included ceftazidime-avibactam (n = 483), cefepime-taniborbactam (n = 4), and cefiderocol (n = 10). This figure summarizes crude mortality across heterogeneous studies and is intended for descriptive comparison only; it does not represent a pooled comparative effect estimate.

#### Interpretation of comparative descriptive findings

Taken together, the systematic review and comparative descriptive analysis revealed a consistent pattern across heterogeneous observational studies: carbapenem therapy was associated with high and variable mortality rates despite low meropenem MICs, whereas treatment with newer agents active against OXA-48-PE was associated with lower and more homogeneous mortality.

Given the heterogeneity of study designs, patient populations, infection types, and outcome definitions, these findings were considered descriptive and hypothesis-generating. To further assess whether the observed differences reflected a true association between treatment strategy and clinical outcome, a formal meta-analysis restricted to studies with direct treatment comparisons was subsequently performed.

### Part 3 – meta-analysis of comparative studies

#### Study selection for meta-analysis

Among the studies identified in the systematic review, seven provided direct, within-study comparisons between carbapenem-based regimens and alternative active therapies in patients with infections caused by OXA-48–PE. Of these, six were human clinical studies and one was an experimental animal study. The primary quantitative meta-analysis was restricted to the six human comparative studies. Across these human studies, a total of 63 patients received carbapenem monotherapy, while 175 patients were treated with alternative active regimens. When the experimental animal study of Mimoz et al. was also considered in sensitivity analysis, the total numbers increased to 159 and 223, respectively. Definitions of clinical failure were broadly comparable across studies but were not strictly identical. In most studies, the extracted endpoint reflected overall treatment failure and generally included all-cause mortality and/or lack of clinical response according to the original study definition.

#### Association between carbapenem therapy and clinical failure

**Figure 2.**
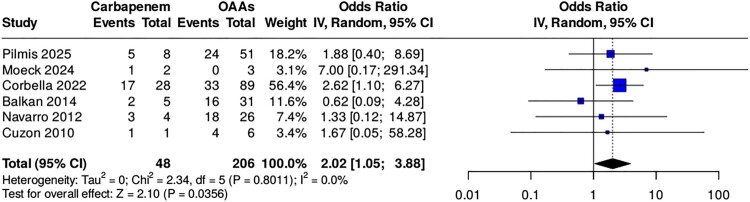
Forest plot of clinical failure in human comparative studies of carbapenem-based versus alternative active regimens for OXA-48–producing Enterobacterales infections.

Meta-analysis of the human comparative studies demonstrated that carbapenem therapy was associated with a higher risk of clinical failure compared with alternative active regimens. In the primary random-effects analysis, the pooled odds ratio (OR) for clinical failure associated with carbapenem therapy was 2.02 (95% confidence interval [CI], 1.05–3.88) (Figure 2). These results support the direction of effect observed across the cohort-level and descriptive literature analyses, although the number of comparative clinical studies remained limited. Study-specific effect estimates and their contribution to the pooled analysis are shown in Figure 2 as a conventional forest plot.

#### Sensitivity analysis

Because one of the seven comparative studies was an experimental animal model, its influence on the pooled estimate was examined separately and was not considered part of the primary clinical quantitative synthesis. Across the six human comparative studies, the total numbers were 63 patients in the carbapenem group and 175 in the alternative-therapy group. When the experimental animal study of Mimoz et al. was also included in sensitivity analysis, these totals increased to 159 and 223, respectively. The overall direction of the association remained unchanged, supporting the robustness of the signal; however, the clinical interpretation of the meta-analysis relies primarily on the human comparative studies.

#### Summary of meta-analytic findings

Taken together, this meta-analysis restricted to comparative studies demonstrated a consistent association between carbapenem therapy and increased risk of clinical failure in OXA-48–PE infections. These findings quantitatively support the signal observed in the French multicenter cohort and the comparative descriptive analysis of the broader literature.

## Discussion

Across this three-level investigation, which combined a national retrospective cohort, a systematic comparative descriptive analysis, and a restricted meta-analysis, carbapenem therapy was consistently associated with unfavourable clinical outcomes in infections caused by OXA-48–producing Enterobacterales. The value of this study lies in the convergence of complementary levels of evidence to address a specific clinical question that remains unresolved in routine practice. Importantly, despite apparent in vitro susceptibility according to EUCAST 2025 clinical breakpoints, carbapenem-based regimens were repeatedly associated with high rates of clinical failure and mortality [15,19,20,21,22].

The French multicenter retrospective cohort provided preliminary clinical evidence. Although limited by sample size and therefore not suitable for inferential statistical comparisons, this cohort showed high failure rates among patients treated with meropenem monotherapy, even when isolates displayed low meropenem MICs. Importantly, carbapenem-treated patients were not restricted to isolates with MICs at the upper boundary of the susceptible range: low meropenem MIC values, including ≤0.25 and 0.5 mg/L, were also observed in this group (Supplementary Table S5). This further highlights a potential disconnect between microbiological categorization and clinical response. These exploratory findings motivated further evaluation at the level of the published literature. In published studies, carbapenem therapy was also predominantly used in patients infected with isolates categorized as susceptible by EUCAST criteria (Supplementary Figure S2) [23].

The systematic review and comparative descriptive analysis demonstrated that this unfavourable signal was not confined to a single cohort. Across heterogeneous observational studies involving different countries, infection types, and patient populations, carbapenem therapy was associated with high and variable crude mortality. In contrast, modern agents active against OXA-48–PE – most notably ceftazidime-avibactam, which accounted for the majority of patients in this class – were associated with lower and more homogeneous crude mortality rates. However, these observations derive from descriptive cross-study comparisons and should not be interpreted as formal pooled comparative effect estimates.

The meta-analysis restricted primarily to human studies with direct treatment comparisons provided quantitative support for this hypothesis. By focusing on within-study comparisons between carbapenem-based regimens and alternative active therapies, this analysis reduced between-study heterogeneity. It also avoided relying on non-human data for the primary clinical interpretation. In the primary random-effects analysis, carbapenem therapy remained associated with an increased risk of clinical failure. However, the number of comparative human studies was limited, and residual confounding cannot be excluded. Sensitivity analysis including the experimental animal study yielded a similar direction of effect.

Several mechanisms may explain the observed discrepancy between *in vitro* susceptibility and poor clinical outcomes with carbapenems in OXA-48–producing Enterobacterales infections. OXA-48-like enzymes confer carbapenem hydrolysis that may not be fully captured by standard MIC testing, particularly at high bacterial inocula. As a result, MIC-based susceptibility categorization may not accurately reflect *in vivo* pharmacodynamic activity. More broadly, MIC-based susceptibility categories are not designed to directly incorporate resistance mechanisms such as carbapenemase production. Rather, they primarily reflect the probability of achieving adequate exposure under defined pharmacokinetic/pharmacodynamic conditions. This is an inherent limitation of current breakpoint frameworks and may partly explain the observed discrepancy between susceptibility categorization and clinical outcome in OXA-48–producing isolates. Experimental studies have demonstrated a pronounced inoculum effect for meropenem against OXA-48 producers, whereas agents such as ceftazidime-avibactam retain activity under similar conditions. Additional factors, including heteroresistance, suboptimal pharmacokinetic exposure in critically ill patients, and the frequent coexistence of other resistance determinants, may further compromise carbapenem efficacy despite low MIC values. It should also be noted that not all OXA-48-like variants are necessarily equivalent in terms of hydrolytic profile or associated resistance background; therefore, our conclusions should be interpreted primarily in relation to the populations represented in the included cohort and clinical studies. These results could also be applied to OXA-48 variants that possess the same carbapenemase activity as OXA-48 (e.g. OXA-181, OXA-162, and OXA-204). For OXA-48 variants with lower carbapenemase activity (e.g. OXA-244 and OXA-484), a true demonstration of the poorer activity of carbapenems might be more difficult to obtain and would likely require more patients. This may also be related to the fact that these variants are mostly produced by E. coli, which is mainly responsible for urinary tract infections, a context in which β-lactams reach very high concentrations at the site of infection. These findings have important clinical implications. OXA-48-like enzymes are now widely disseminated internationally, so the relevance of these observations extends beyond Europe. The 2025 EUCAST clinical breakpoints define carbapenem susceptibility on the basis of MIC and expected exposure, rather than on the presence or absence of a specific resistance mechanism. As a result, isolates producing OXA-48 may remain categorized as susceptible despite a mechanism that can still compromise clinical response, particularly in severe infections or high-inoculum settings. In parallel, several infectious diseases societies discourage carbapenem monotherapy for carbapenemase-producing Enterobacterales and recommend prioritizing newer β-lactam/β-lactamase inhibitor combinations when available [3,4,25–28]. Our findings are aligned with this emerging expert guidance and support caution when interpreting carbapenem susceptibility solely on the basis of MIC values in OXA-48-producing isolates. However, given the observational and partly descriptive nature of the available evidence, these findings should be interpreted as supportive of such guidance rather than as direct evidence for guideline change.

This study has several limitations. Most available clinical data derive from retrospective observational studies. They are therefore subject to confounding by indication and to heterogeneity in patient severity, infection types, and outcome definitions. In addition, although the meta-analysis focused on the closest available endpoint reflecting overall treatment failure, the exact definitions of clinical failure were not fully identical across studies, which may have influenced the pooled estimate. In the French cohort, treatment allocation was not randomized. Carbapenems may therefore have been used in patients with different severity profiles or in different clinical settings. Although we added descriptive baseline comparisons according to definitive treatment group, exploratory Fisher’s exact tests for selected categorical comparisons, and a descriptive stratification of 30-day mortality according to infection source, the small sample size precluded robust adjustment for these imbalances. It also limited the interpretability of crude outcome differences between treatment groups. Data on carbapenem dosing strategies, including prolonged infusion or high-dose regimens, were inconsistently reported and could not be analyzed. Finally, although the meta-analysis was restricted primarily to human comparative studies, residual confounding cannot be fully excluded, as within-study comparisons reduce but do not eliminate confounding by indication.

Nevertheless, the convergence of findings across three complementary analytical levels strengthens the overall conclusions.

## Conclusion

In this three-level analysis combining a national retrospective cohort, a systematic comparative review, and a restricted meta-analysis, carbapenem therapy was consistently associated with unfavourable clinical outcomes in infections caused by OXA-48–PE. Importantly, this association was observed despite low carbapenem minimum inhibitory concentrations and across diverse study designs and patient populations.

While carbapenems have historically been used to treat OXA-48–producing isolates based on MIC-based susceptibility criteria, the present findings challenge the clinical reliability of this approach. In contrast, newer agents active against OXA-48-PE, particularly β-lactam/β-lactamase inhibitor combinations, were associated with more favourable and consistent outcomes.

Taken together, these results support carbapenem-sparing treatment strategies for OXA-48–PE infections whenever effective alternatives are available.

## Supplementary Material

Supplementary Table S3.docx

Supplementary Table S2.docx

Supplementary Table S4.docx

Supplementary Figure S1.docx

Supplementary Figure S2docx.docx

Supplementary Table S5.docx

Supplementary Table S1.docx
