# Supplementary material for: Clinical outcomes of carbapenem therapy in OXA-48–producing Enterobacterales infections: a French multicentre cohort, systematic review, and meta-analysis
Source: Emerg Microbes Infect. 2026 May 7;15(1):2671518. doi: 10.1080/22221751.2026.2671518 (PMC13188539; doi:10.1080/22221751.2026.2671518)
Supplement: Supplementary Table S3.docx [file TEMI_A_2671518_SM3593.docx]

**Supplementary Table S3**. Exploratory 30-day mortality according to infection source and definitive treatment group in the French cohort

| **Infection source** | **Carbapenem 30-day mortality, x/N (%)** | **Ceftazidime-avibactam 30-day mortality, x/N (%)** | **Other active alternatives 30-day mortality, x/N (%)** | **All alternatives pooled 30-day mortality, x/N (%)** |
| --- | --- | --- | --- | --- |
| Urinary tract infection | 1/2 (50.0) | 2/8 (25.0) | 1/3 (33.3) | 3/11 (27.3) |
| Intra-abdominal infection | 1/1 (100.0) | 3/5 (60.0) | 3/6 (50.0) | 6/11 (54.5) |
| Respiratory tract infection | 0/1 (0) | 3/4 (75.0) | 3/6 (50.0) | 6/10 (60.0) |
| Catheter-related infection | 1/1 (100.0) | 2/4 (50.0) | 2/3 (66.7) | 4/7 (57.1) |
| Skin and soft tissue infection | 1/2 (50.0) | 1/3 (33.3) | 2/2 (100.0) | 3/5 (60.0) |
| Other infection | 1/1 (100.0) | 1/4 (25.0) | 1/3 (33.3) | 2/7 (28.6) |
