# Supplementary material for: Clinical outcomes of carbapenem therapy in OXA-48–producing Enterobacterales infections: a French multicentre cohort, systematic review, and meta-analysis
Source: Emerg Microbes Infect. 2026 May 7;15(1):2671518. doi: 10.1080/22221751.2026.2671518 (PMC13188539; doi:10.1080/22221751.2026.2671518)
Supplement: Supplementary Table S2.docx [file TEMI_A_2671518_SM3592.docx]

## **Supplementary Table S2.** Treatment regimens and clinical outcomes in 43 patients with OXA-48 bloodstream infections

| Treatment regimen | Subgroup | n/N | Failure rate (%) |
| --- | --- | --- | --- |
| Meropenem | All | 4/7 | 57.1 |
|  | MIC ≤2 mg/L | 3/6 | 50.0 |
|  | MIC 16 mg/L | 1/1 | 100.0 |
| Other active agents | All | 13/36 | 36.1 |
|  | Ceftazidime-avibactam | 10/28 | 35.7 |
|  | Cefepime | 1/4 | 25.0 |
|  | Trimethoprim-sulfamethoxazole | 0/2 | 0.0 |
|  | Ceftazidime | 1/1 | 100.0 |
|  | Colistin | 1/1 | 100.0 |

Data are number of failures per total patients (%). MIC, minimum inhibitory concentration. One patient in the meropenem group had an isolate with MIC=16 mg/L; all others had MIC ≤2 mg/L. OAA = Other active agents.
