# Supplementary material for: Clinical outcomes of carbapenem therapy in OXA-48–producing Enterobacterales infections: a French multicentre cohort, systematic review, and meta-analysis
Source: Emerg Microbes Infect. 2026 May 7;15(1):2671518. doi: 10.1080/22221751.2026.2671518 (PMC13188539; doi:10.1080/22221751.2026.2671518)
Supplement: Supplementary Table S4.docx [file TEMI_A_2671518_SM3591.docx]

**Supplementary Table S4.** Alternative therapies used in selected studies

| **Author [ref]** | **Year** | **Country** | **Study type** | **Design** | **Total number of subjects** | **Carbapenem monotherapy group** | **Alternatives group (including multiple therapy without carbapenem)** | | | | | | | | | | | | **NOS** | **ROB2** |
| --- | --- | --- | --- | --- | --- | --- | --- | --- | --- | --- | --- | --- | --- | --- | --- | --- | --- | --- | --- | --- |
|  |  |  |  |  |  | n | n | FEP/TAN | TGC | AG | CS | FEP | CAZ | CZA | TMP/SMX | FQ | FOS | N/A |  |  |
| Internal cohort | 2025 | France | Human | Retrospective | 43 | 7 | 36 | 0 | 0 | 0 | 1 | 4 | 1 | 28 | 2 | 0 | 0 | 0 | 7 | - |
| Moeck ^28^ | 2024 | USA | Human | Prospective | 5 | 2 | 3 | 3 | 0 | 0 | 0 | 0 | 0 | 0 | 0 | 0 | 0 | 0 | - | Low risk of bias |
| Corbella^15^ | 2022 | Spain | Human | Retrospective | 117 | 44 | 73 | 0 | 32 | 23 | 14 | 0 | 0 | 2 | 9 | 0 | 0 | 13 | 8 | - |
| Balkan^19^ | 2014 | Turkey | Human | Retrospective | 36 | 5 | 31 | 0 | 0 | 9 | 24 | 0 | 0 | 0 | 0 | 0 | 0 | 7 | 7 | - |
| Navarro- San Francisco^9^ | 2012 | Spain | Human | Prospective | 30 | 4 | 26 | 0 | 15 | 5 | 18 | 0 | 0 | 0 | 0 | 0 | 7 | 5 | 7 | - |
| Mimoz^20^ | 2012 | France | Animal | Prospective | 240 | 96 | 48 | 0 | 0 | 0 | 0 | 0 | 48 | 0 | 0 | 0 | 0 | 0 | - | - |
| Cuzon^29^ | 2010 | France | Human | Retrospective | 7 | 1 | 6 | 0 | 0 | 2 | 5 | 0 | 0 | 0 | 0 | 1 | 0 | 0 | 6 | - |

AG:Aminoglycosides, CAZ: Ceftazidime, CS: colistin, CZA: ceftazidime/avibactam, FEP: Cefepime, FEP/TAN: Cefepime:taniborbactam, FOS: Fosfomycin, FQ: fluoroquinolones, N/A: Not available, TGC: Tigecycline, TMP/SMX: Trimethoprim/sulfamethoxazole
