# Supplementary material for: Clinical outcomes of carbapenem therapy in OXA-48–producing Enterobacterales infections: a French multicentre cohort, systematic review, and meta-analysis
Source: Emerg Microbes Infect. 2026 May 7;15(1):2671518. doi: 10.1080/22221751.2026.2671518 (PMC13188539; doi:10.1080/22221751.2026.2671518)

**Supplementary Figure S1.** Distribution of meropenem minimum inhibitory concentrations (MICs) among OXA-48-producing Enterobacterales isolates. Meropenem or active alternative regimen used to treat the infection were indicated in blue and green, respectively. Dotted lines corresponded to clinical breakpoints of meropenem (in green : S to I, in red : I to R) according to EUCAST guidelines.


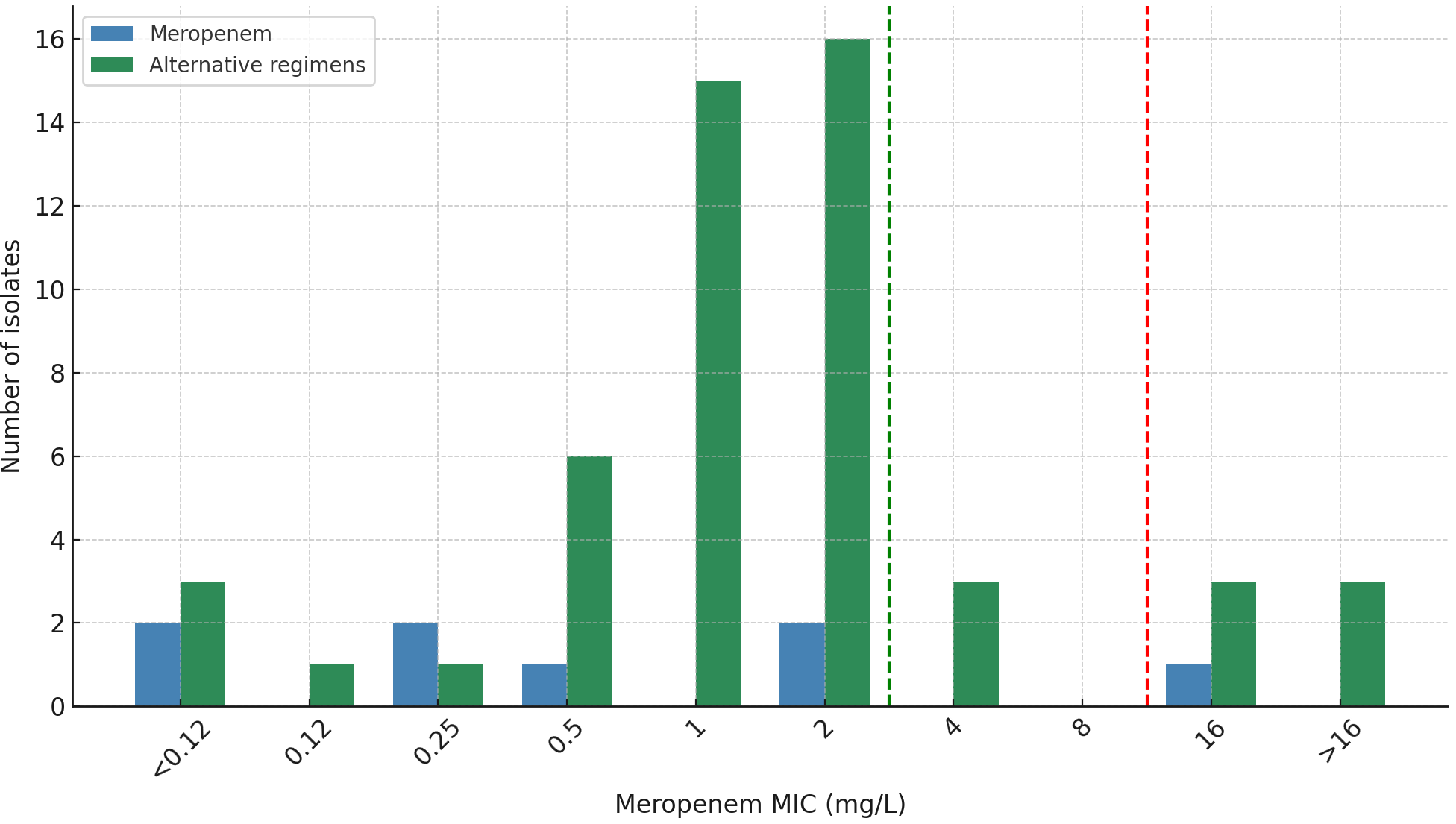

Supplement: Supplementary Figure S1.docx [file TEMI_A_2671518_SM3590.docx]
