# Supplementary material for: Clinical outcomes of carbapenem therapy in OXA-48–producing Enterobacterales infections: a French multicentre cohort, systematic review, and meta-analysis
Source: Emerg Microbes Infect. 2026 May 7;15(1):2671518. doi: 10.1080/22221751.2026.2671518 (PMC13188539; doi:10.1080/22221751.2026.2671518)
Supplement: Supplementary Figure S2docx.docx [file TEMI_A_2671518_SM3589.docx]

**Supplementary Figure S2**. MIC distribution of meropenem among OXA-48-producing isolates according to treatment regimen
