# Supplementary material for: Clinical outcomes of carbapenem therapy in OXA-48–producing Enterobacterales infections: a French multicentre cohort, systematic review, and meta-analysis
Source: Emerg Microbes Infect. 2026 May 7;15(1):2671518. doi: 10.1080/22221751.2026.2671518 (PMC13188539; doi:10.1080/22221751.2026.2671518)
Supplement: Supplementary Table S5.docx [file TEMI_A_2671518_SM3588.docx]

**Supplementary Table S5.** Meropenem MIC distribution according to definitive treatment group in the French cohort

| **Meropenem MIC (mg/L)** | **Total**  **(n=59)** | **Carbapenem monotherapy (n=8 )** | **Ceftazidime-avibactam (n=28 )** | **Other active alternatives (n=23)** | **All alternative active therapy pooled**  **(n=51)** |
| --- | --- | --- | --- | --- | --- |
| ≤0.25 | 9 (15.2) | 3 (37.5) | 3 (10.7) | 3 (13.1) | 6 (11.8) |
| 0.5 | 8 (13.6) | 1 (12.5) | 4 (14.3) | 3 (13.1) | 7 (13.7) |
| 1 | 14 (23.7) | 0 (0) | 8 (28.5) | 6 (26.2) | 14 (27.5) |
| 2 | 18 (30.5) | 3 (37.5) | 8 (28.5) | 7 (30.4) | 15 (29.4) |
| 4 | 3 (5.1) | 0 (0) | 2 (7.2) | 1 (4.3) | 3 (5.9) |
| 8 | 1 (1.7) | 0 (0) | 1 (3.6) | 0 (0) | 1 (1.9) |
| 16 | 3 (5.1) | 1 (12.5) | 1 (3.6) | 1 (4.3) | 2 (3.9) |
| >16 | 3 (5.1) | 0 (0) | 1 (3.6) | 2 (8.7) | 3 (5.9) |

Data are presented as number of isolates (% within treatment group). MIC, minimum inhibitory concentration. Percentages may not total 100 because of rounding.
