# Supplementary material for: Clinical outcomes of carbapenem therapy in OXA-48–producing Enterobacterales infections: a French multicentre cohort, systematic review, and meta-analysis
Source: Emerg Microbes Infect. 2026 May 7;15(1):2671518. doi: 10.1080/22221751.2026.2671518 (PMC13188539; doi:10.1080/22221751.2026.2671518)
Supplement: Supplementary Table S1.docx [file TEMI_A_2671518_SM3587.docx]

# **Supplementary Table S1**. Complete Electronic Search Strategies

| **Database** | **Search Strategy** |
| --- | --- |
| PubMed | ("OXA-48" OR "OXA 48" OR "OXA48") AND ("*Enterobacterales*" OR "*Enterobacteriaceae*" OR "*Klebsiella pneumoniae*" OR "*Escherichia coli*") AND ("carbapenem monotherapy" OR "carbapenem treatment" OR "meropenem" OR "imipenem" OR "ertapenem" OR "doripenem" OR “ceftazidime/avibactam” OR “cefiderocol” OR “colistin” OR “tigecycline” OR “polymyxin B” OR “cefepime/enmetazobactam”) AND ("clinical failure" OR "therapeutic failure" OR "treatment failure" OR "mortality" OR "outcome" OR "survival" OR "microbiological eradication" OR "bacteriological eradication") |
| Embase | (“OXA-48” OR “OXA 48” OR “OXA48”) AND (“*Enterobacterales*” OR “*Enterobacteriaceae*” OR “*Klebsiella pneumoniae*” OR “*Escherichia coli*”) AND (“carbapenem monotherapy” OR “carbapenem treatment” OR "meropenem" OR "imipenem" OR "ertapenem" OR "doripenem" OR “ceftazidime/avibactam” OR “cefiderocol” OR “colistin” OR “tigecycline” OR “polymyxin B” OR “cefepime/enmetazobactam”) AND (“clinical failure” OR “therapeutic failure” OR “treatment failure” OR “mortality” OR “outcome” OR “survival” OR “microbiological eradication” OR “bacteriological eradication”) |
| Web of Science | TS=("OXA-48" OR "OXA 48" OR "OXA48") AND TS=("*Enterobacterales*" OR "*Enterobacteriaceae*" OR "*Klebsiella pneumoniae*" OR "*Escherichia coli*") AND TS=("carbapenem monotherapy" OR "carbapenem treatment" OR "meropenem" OR "imipenem" OR "ertapenem" OR "doripenem" OR “ceftazidime/avibactam” OR “cefiderocol” OR “colistin” OR “tigecycline” OR “polymyxin B” OR “cefepime/enmetazobactam”) AND TS=("clinical failure" OR "therapeutic failure" OR "treatment failure" OR "mortality" OR "outcome" OR "survival" OR "microbiological eradication" OR "bacteriological eradication") |
